# Anti-*Staphylococcus aureus* Activity and Structural Characterization of Rationally Designed Peptides

**DOI:** 10.3390/antibiotics14050437

**Published:** 2025-04-26

**Authors:** Lorenza Artesani, Mariana Gallo, Laura Giovati, Francesca Maria Bisignano, Elena Ferrari, Lara M. Castronovo, Stefania Conti, Francesco Santoro, Thelma A. Pertinhez, Tecla Ciociola

**Affiliations:** 1 Laboratory of Microbiology and Virology, Department of Medicine and Surgery, University of Parma, 43125 Parma, Italy; lorenza.artesani@unipr.it (L.A.); laura.giovati@unipr.it (L.G.); francescamaria.bisignano@unipr.it (F.M.B.); tecla.ciociola@unipr.it (T.C.); 2 Laboratory of Biochemistry and Metabolomics, Department of Medicine and Surgery, University of Parma, 43125 Parma, Italy; mariana.gallo@unipr.it (M.G.); elena.ferrari@unipr.it (E.F.); thelma.pertinhez@unipr.it (T.A.P.); 3 Microbiome Research Hub, University of Parma, 43124 Parma, Italy; 4 Laboratory of Molecular Microbiology and Biotechnology, Department of Medical Biotechnologies, University of Siena, 53100 Siena, Italy; lara.castronovo@unisi.it (L.M.C.); santorof@unisi.it (F.S.)

**Keywords:** antimicrobial peptides, *Staphylococcus aureus*, MRSA, VRSA, structural characterization, circular dichroism, nuclear magnetic resonance spectroscopy

## Abstract

**Background/Objectives**: Microbial infections represent a significant threat to public health due to the emergence and spread of antimicrobial resistance. Adjunctive and alternative therapeutic strategies are explored to tackle this issue, including the use of natural or synthetic antimicrobial peptides. Previous research showed that antibody-derived peptides possess antimicrobial, antiviral, and immunomodulatory properties. This study aimed to characterize newly designed antibody-derived peptides and evaluate their effectiveness against representative strains of *Staphylococcus aureus*, including drug-resistant isolates. **Methods**: Colony-forming unit assays and confocal microscopy studies were performed to evaluate peptide activity against planktonic microbial cells. Cytotoxicity tests were performed on THP-1 human monocytic cells. Circular dichroism (CD) and nuclear magnetic resonance (NMR) were employed for the conformational characterization of peptides. **Results**: The half-maximal effective concentrations of the peptides against bacterial reference strains and drug-resistant isolates ranged from 0.17 to 18.05 µM, while cytotoxic effects were not observed against mammalian cells. A killing kinetics analysis and observation by confocal microscopy of the interaction between peptides and bacteria suggested a mechanism of action involving membrane perturbation. CD studies showed that all peptides predominantly exhibit a random coil arrangement in aqueous solution. NMR spectroscopy revealed that the most active peptide adopts a helical conformation in the presence of membrane mimetics. **Conclusions**: The structural characterization and evaluation of the newly designed peptides’ antimicrobial activity may lead to the selection of a candidate to be further studied to develop an alternative treatment against microbial infections caused by drug-resistant strains.

## 1. Introduction

Bacterial infections still pose a significant challenge to global health. The introduction of antibacterial drugs into clinical practice had a crucial impact on the prognosis of many infectious diseases. However, this was soon followed by the emergence and spread of resistant bacterial strains [1,2]. According to the World Health Organization (WHO), antimicrobial resistance (AMR) is one of the top global public health and development threats. The excessive and inappropriate use of antimicrobials in humans, animals, and plants largely contributed to the worsening of the problem of AMR [3]. In 2021, it was estimated that approximately 4.71 million deaths were related to bacterial infections, of which 1.14 million were attributable to drug-resistant bacteria [4], with slightly decreased values compared to the previous study [5].

*Staphylococcus aureus* is one of the leading causes of healthcare-associated and community-acquired infections worldwide, causing diseases ranging from mild skin infections to fatal multiorgan failure [6]. It is also involved in livestock-associated infections [7]. The incidence of staphylococcal infections is increased in the presence of predisposing conditions, such as diabetes, dialysis, the use of invasive medical devices (peripheral and/or central venous catheters), drug use, and other infections (e.g., HIV) [8,9]. Bacteremia caused by *S. aureus* has an annual incidence rate of more than 50 cases per 100,000 inhabitants and, despite effective antibacterial therapies, mortality rates approach 20–30% in developed countries [10,11]. The very high pathogenic potential of *S. aureus* derives from a variety of structural components and, even more significantly, from the numerous soluble substances (exotoxins and exoenzymes) it characteristically produces [12,13]. The virulence factors are involved in the induction of the inflammatory response, adhesion, invasion, immune system evasion, cell lysis, and in promoting bacterial survival and dissemination [12,14,15].

Since the onset of the antibiotic era, *S. aureus*-resistant strains played an important role. Methicillin-resistant *S. aureus* (MRSA) is currently listed among the high-priority pathogens according to the WHO [16], in line with its high impact on public health and its prevalence in high-income countries, where it represents one of the most widespread drug-resistant pathogens [17]. Glycopeptides have become a common and often mandatory choice for treating MRSA infections in hospitalized patients since the early 1960s. It is therefore understandable the particular concern about the emergence of MRSA strains that initially had a low level of resistance to glycopeptides [18] and then became highly resistant to these drugs (vancomycin-resistant *S. aureus*, VRSA) [19], and in recent years, also became resistant to linezolid, which is considered the last line of defense against *S. aureus* infections [20]. The Global Burden of Disease study reported that in high-income countries, approximately 50% of deaths attributed to AMR are linked to two pathogens: *S. aureus* and *Escherichia coli* [5].

To address the AMR threat, adjunctive and/or alternative therapeutic strategies are envisaged [21,22,23,24]. Among the various approaches, natural or synthetic antimicrobial peptides (AMPs) arouse great interest as potential anti-infective drugs [25,26,27,28]. Natural AMPs are found in various organisms as crucial components of the host’s innate immune system (also called host defense peptides). They are characterized by broad-spectrum activity against microorganisms, viruses, and even cancer cells. Furthermore, their distinctive mechanism of action reduces the likelihood of developing drug resistance [27,29,30,31,32].

The present study is based on the L18R peptide, the translated product of the immunoglobulin J gene (locus heavy, IGHJ2) [33]. L18R exhibits antimicrobial activity against yeasts and bacteria, including strains that are resistant to drugs and/or form biofilms. L18R was demonstrated to possess a therapeutic effect in an experimental model of systemic infection by *Candida albicans* in larvae of *Galleria mellonella* [33]. Moreover, it showed promising efficacy for the treatment of polymicrobial infections in chronic wounds [34]. L18R also proved to be effective against *Enterococcus faecalis*. This bacterium is responsible for the formation of resistant biofilms at the level of the dentinal walls of the root canal, causing endodontic infections [35]. Here, we explored the potential enhancement of the biological properties of L18R by generating a set of rationally modified L18R-derived peptides. These new molecules were tested for their toxicity against THP-1 human monocytic cells and in vitro antibacterial activity against *S. aureus* strains, including drug-resistant isolates. The conformational characterization of the investigated peptides and their interactions with bacterial components and mimetics were analyzed by circular dichroism (CD) measurements. Focusing on the peptide with the best killing performance, the mechanism of action was investigated via confocal laser scanning microscopy. Additionally, we performed structural characterization at a residue level using nuclear magnetic resonance spectroscopy (NMR). Overall, a comprehensive functional and structural analysis of L18R-derived peptides was provided. Future studies, including in vivo experiments, will contribute to a more accurate assessment of their potential for therapeutic use.

## 2. Results

### 2.1. Peptide Design

The design of the new molecules was based on the L18R peptide (Table 1), which served as a lead molecule due to its promising activity against yeasts and bacteria [33,34,35]. Recognizing that the positive charges on AMPs are critical for their interaction with bacterial surfaces [36], all the designed peptides retained the same number of positive charges (2+) as the parent L18R peptide. Modifications on key amino acid residues were introduced to explore their influence on peptide activity. Specifically, alanine replaced the α-helix breaker proline in L18R-AA to promote helix formation. In the L18R-10A, alanine substituted the tryptophan residue at position 10 to remove its bulky side chain. Alanine substituted cysteine in L18R-15A to assess the effect of this residue, known for its ability to form peptide dimers [37]. Additionally, arginine at position 5 and leucine at position 7 were exchanged in L18R-LR to create a hydrophobic patch at the N-terminus, which may be important for interacting with the bacterial surfaces.

### 2.2. Cytotoxicity

**Figure 1:**
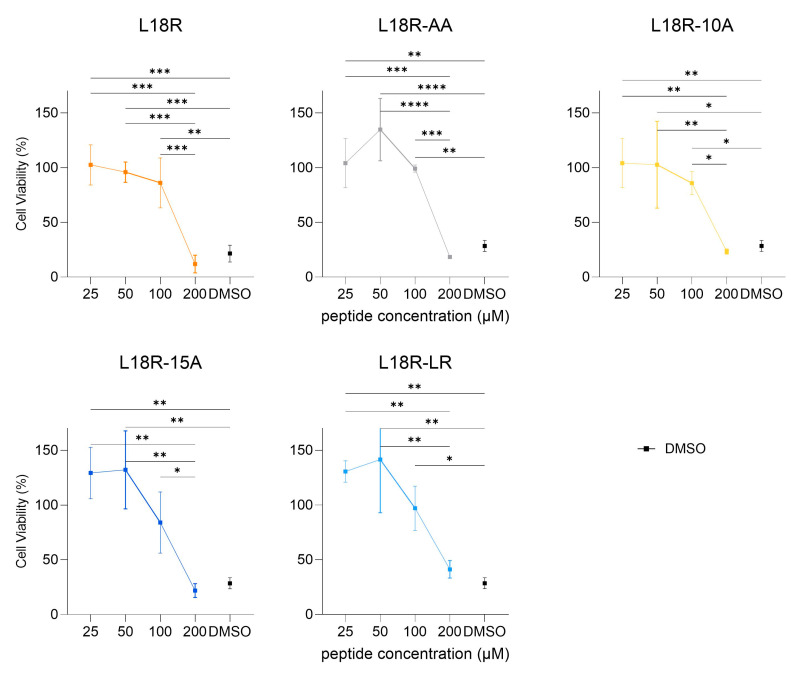
In vitro cytotoxic activity on THP-1 cells in response to increasing concentrations of the investigated peptides. DMSO at the same concentration as in 200 µM peptide solutions (about 2%) was used as control (black squares). Statistical significance of differences in cell viability was assessed with one-way ANOVA, followed by post hoc analysis with Tukey’s test correction (* *p* < 0.05, ** *p* < 0.01, *** *p* < 0.001, and **** *p* < 0.0001).

The cytotoxicity of L18R and its derivatives was assessed against THP-1 human monocytic cells. None of the investigated peptides showed significant toxicity at concentrations of up to 100 µM. At 200 µM, cell viability decreased to values comparable to those obtained using the DMSO vehicle alone (Figure 1).

### 2.3. In Vitro Antibacterial Activity

**Figure 2:**
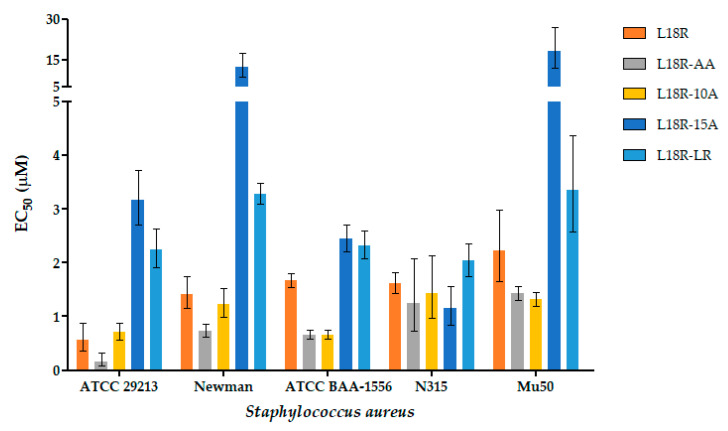
In vitro antibacterial activity of selected peptides against *S. aureus* strains. Activity is expressed as EC_50_ values with 95% confidence intervals.

The bactericidal activity of the L18R derivatives against the *S. aureus* drug-susceptible reference strains, as well as MRSA and MRSA/VRSA isolates, was evaluated in vitro using a colony-forming unit (CFU) assay. As shown in Figure 2 and Table S1, all the peptides were effective against all the tested strains, with a half-maximal effective concentration (EC_50_) ranging from 0.17 to 18.05 μM. Compared to the parental L18R peptide, L18R-LR and L18R-15A generally showed lower activity, while L18R-AA and L18R-10A exhibited the highest activity against all tested strains, except for *S. aureus* N315. Against this strain, the activity of all derivatives was comparable. Additionally, L18R-AA was more effective than L18R-10A against *S. aureus* ATCC 29213 and *S. aureus* Newman, while both peptides had comparable activity against the other tested strains.

**Figure 3:**
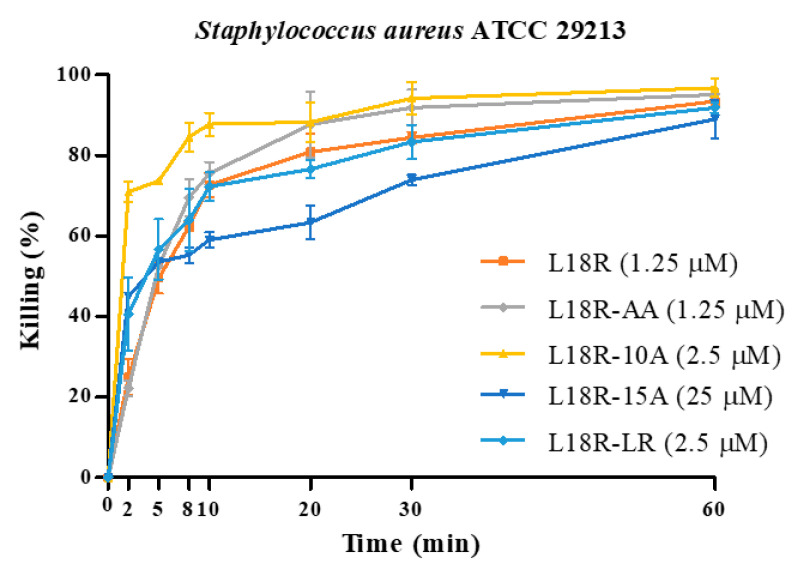

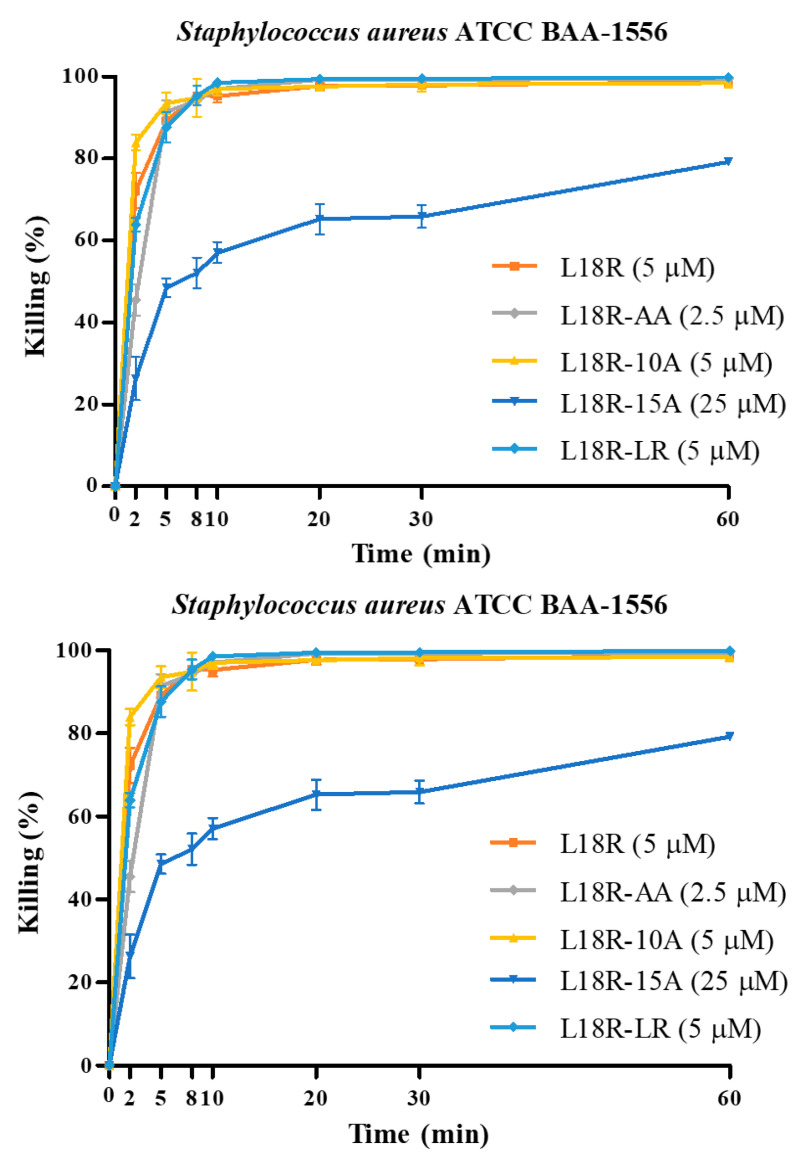
Time kinetics of in vitro activity of peptides against *S. aureus* strains. Peptide concentrations were equal to half of those able to determine 100% killing in CFU assay. Activity is expressed as percentage of killing; data reported are shown as mean values ± SD.

The killing kinetics of L18R and its derivatives against selected *S. aureus* strains (the drug-susceptible ATCC 29213 reference strain, the MRSA ATCC BAA-1556 strain, and the MRSA/VRSA Mu50 strain) were determined using peptides at concentrations equal to half of those able to determine 100% killing in the CFU assay. The bactericidal activity of peptides was rapid against the investigated strains (Figure 3). In fact, after only 10 min of treatment, the killing ranged from 72.3% to 87.8% for *S. aureus* ATCC 29213, from 95.3% to 98.5% for *S. aureus* ATCC BAA-1556, and from 67.6% to 93.0% for *S. aureus* Mu50 for all the peptides studied, except for L18R-15A, whose killing ranged in lower intervals. The time-kill kinetics observed for the derived peptides is compatible with a membranolytic mechanism of action.

### 2.4. Confocal Laser Scanning Microscopy (CLSM) Study

**Figure 4:**
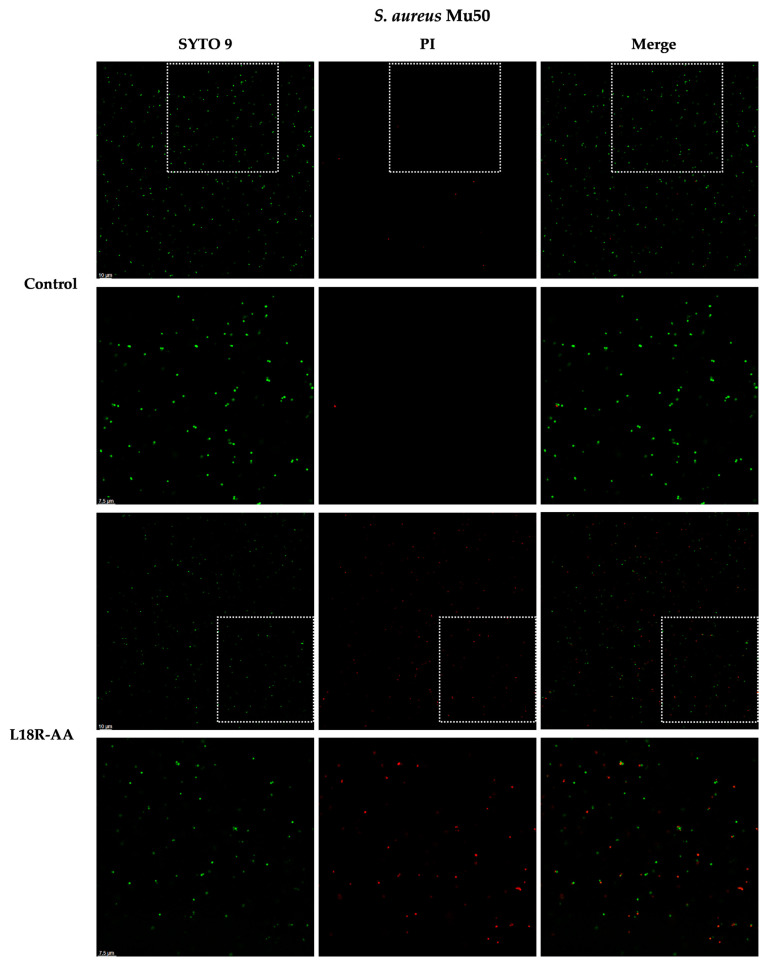
CLSM images of viable *S. aureus* Mu50 cells treated with 100 µM L18R-AA peptide. Bacterial cells were labeled with SYTO 9 and propidium iodide (PI) after 5 min of treatment with or without (control) peptide. Detail of field highlighted by dotting is shown at higher magnification in row below. Bars, 10 and 7.5 µm, respectively.

CLSM studies were conducted with the L18R-AA peptide, selected for its relevant antibacterial activity against all tested bacterial strains. After incubation for 5 min with L18R-AA, bacterial cells were labeled with the fluorophores SYTO 9 and propidium iodide (PI) to allow a qualitative analysis of cell viability. While SYTO 9 enters viable cells, PI is internalized only in the presence of irreversible membrane damage, resulting in cell death. Figure 4 shows representative images of *S. aureus* Mu50 cells (MRSA/VRSA strain) acquired after 5 min of treatment with or without (control) the L18R-AA peptide. In comparison to the control sample, the peptide caused the death of bacterial cells. This behavior is attributable to a rapid, irreversible permeabilization of the cell membrane and confirms the membranolytic mechanism of action hypothesized in the time-killing tests. The same behavior was observed for the other bacterial strains examined (the drug-susceptible ATCC 29213 reference strain and the MRSA ATCC BAA-1556 strain) (Figures S1 and S2).

### 2.5. Structural Characterization of Investigated Peptides

The structural characterization of the investigated peptides and their interactions with the bacterial component and mimetics were determined in CD and NMR studies.

#### 2.5.1. Circular Dichroism Analysis and Interaction with System Mimetics

The global structural characteristics of the peptides were analyzed, using CD spectroscopy, in aqueous solution alone or in the presence of membrane mimetics and bacterial surface components to evaluate their potential interaction with bacteria.

**Figure 5:**
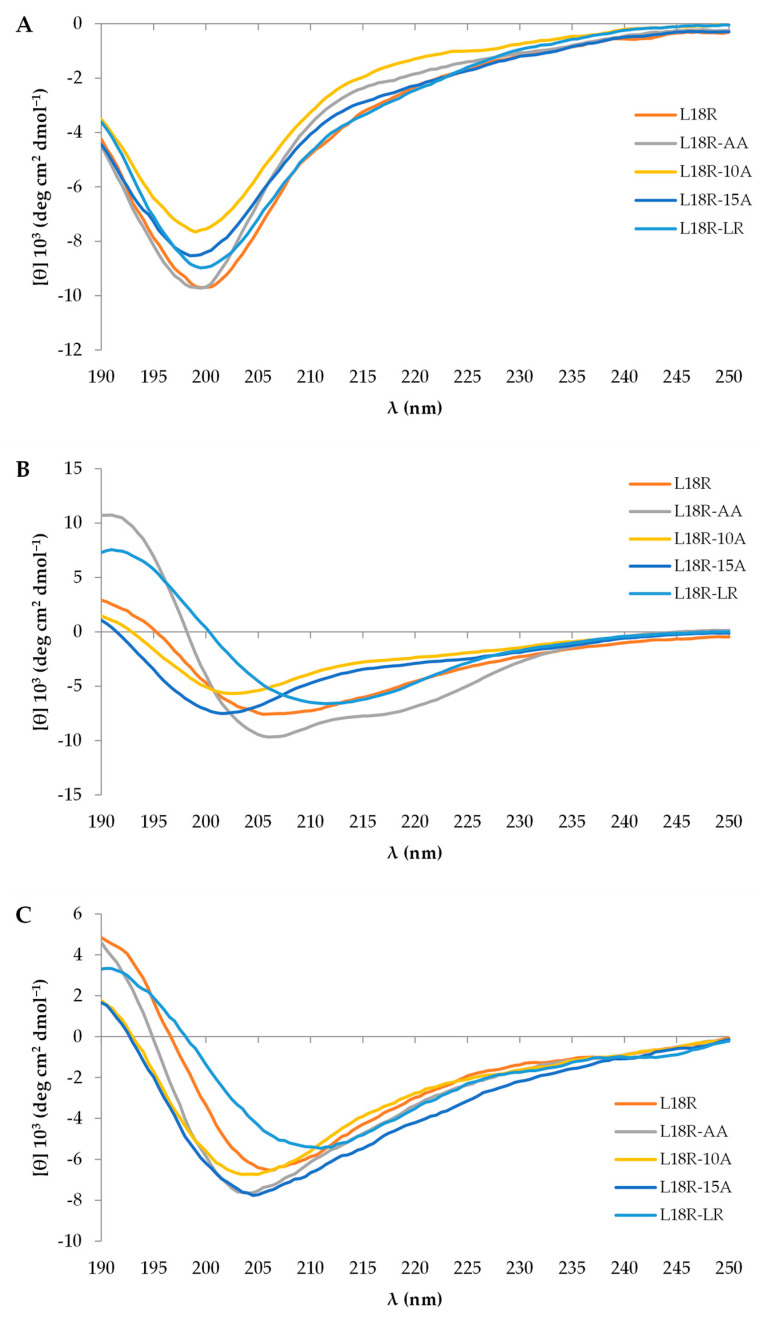
Far-UV CD spectra of peptide samples in water (**A**), in 50 mM sodium dodecyl sulphate (SDS, (**B**)), and in 0.02% lipoteichoic acid (LTA, (**C**)) after 3 weeks from their solubilization.

CD spectra were acquired using aqueous samples at three time points: freshly dissolved, 3 weeks, and 3 months after solubilization. At all of these time points, the peptides displayed a random coil conformation characterized by a single negative band around 200 nm. Figure 5A illustrates the spectra obtained after 3 weeks.

In sodium dodecyl sulphate (SDS), which serves as a mimetic of bacterial anionic membranes, the L18R-AA peptide adopted an α-helix conformation (Figure 5B) with the typical negative bands at 206 and 218 nm, along with a positive band at 191 nm. L18R and L18R-10A showed a helical tendency, as evidenced by a decrease in the 200 nm band, typical of random coil conformation, and a positive increase in the 190 nm band. The L18R-LR peptide demonstrated different behavior, acquiring a β-sheet conformation.

In the presence of lipoteichoic acid (LTA), a structural component of the cell wall of Gram-positive bacteria (Figure 5C), all peptides tended to acquire some secondary structure. Specifically, the L18R-LR peptide exhibited a β-sheet conformation.

**Figure 6:**
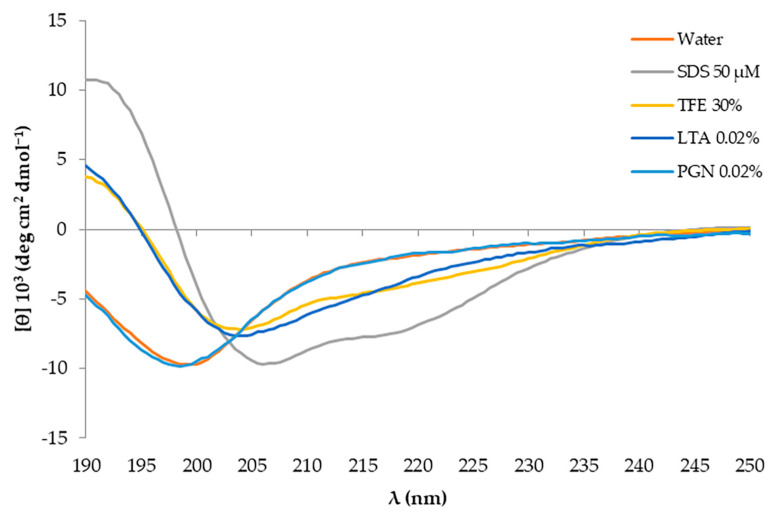
Far-UV CD spectra of L18R-AA peptide in water and in 50 mM sodium dodecyl sulphate (SDS), 0.02% lipoteichoic acid (LTA), 30% 2,2,2-trifluoroethanol (TFE), and 0.02% peptidoglycan (PGN) after 3 weeks from its solubilization.

The propensity of the L18R-AA peptide to form an α-helical structure was further confirmed in the presence of 30% 2,2,2-trifluoroethanol (TFE) and LTA (Figure 6). Finally, exposure to peptidoglycan (PGN), another structural component of the bacterial cell wall, did not significantly influence the structure of the L18R-AA peptide.

#### 2.5.2. Structural Characterization of L18R-AA by Nuclear Magnetic Resonance

We preliminarily assessed the antibacterial activity of the reduced and oxidized forms of L18R-AA. As both states of the peptide exhibited similar activity levels, we chose to perform an NMR structural analysis on the reduced form of L18R-AA.

**Figure 7:**
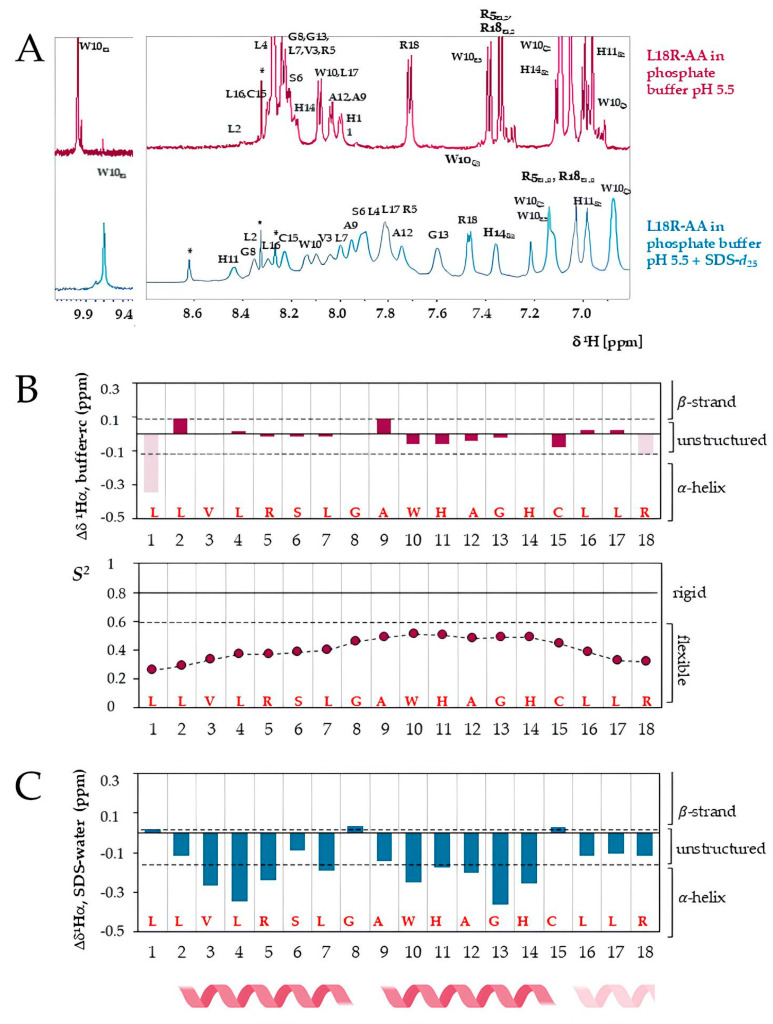
(**A**) L18R-AA interacts with SDS micelles. The 1D-^1^H NMR spectra of 2 mM L18R-AA in 50 mM phosphate buffer at pH 5.5 at 25 °C (**top**) and in the presence of 50 mM SDS-*d*_25_ micelles (**bottom**). (**B**) L18R-AA in buffer is non-structured and flexible. Secondary H_α_ chemical shifts (**top**) and the order parameter (S^2^) were calculated from the backbone chemical shifts using the TALOS-N program (**bottom**) as a function of the residue number. (**C**) The *α*-helix conformation is stabilized in the presence of SDS micelles. H_α_ chemical shift differences in L18R-AA in SDS-*d*_25_ vs. buffer are reported as a function of the peptide sequence. The helical regions are indicated at the bottom. * indicates buffer impurities.

The 1D-^1^H spectrum of the reduced L18R-AA peptide in phosphate buffer (Figure 7A, top spectrum) showed sharp peaks and low chemical shift (CS) dispersion, indicating that the peptide in solution is flexible and unstructured, which agrees with the CD data. The analysis of the 2D spectra (^1^H-^1^H ROESY, ^1^H-^1^H TOCSY, and ^1^H-^13^C HSQC spectra) allowed for the resonance assignment of all the protons and the carbons bonded to hydrogen (Figure S3, Table S2). The CS value of the ^13^C_β_ signal of the Cys15 resonating at 27.8 ppm confirmed the reduced state of the peptide (Figure S3B) [38].

Deviations of backbone CSs from standard random coil values, the secondary shifts, depend on the secondary structure propensity of peptides and proteins [39]. Accordingly, we based the structural characterization of L18R-AA on the deviations of the observed CS from the values predicted for the peptide in random conformation. L18R-AA in buffer is predominantly in random coil conformation, as indicated by the observed low secondary shift values of H_α_, C_α_, and C_β_ (in Figure 7B, the top panel depicts only the H_α_ secondary shifts). The order parameter (S^2^), which reflects the peptide’s flexibility, was calculated from the backbone CSs. S^2^ values range from 0 to 1; regions of flexibility exhibit S^2^ values lower than 0.6, whereas rigid regions in peptide structures typically have values around 0.80–0.85. For the L18R-AA peptide, S^2^ values were below 0.6 for all amino acids (Figure 7B, bottom panel).

The spectrum in Figure 7A (bottom) shows broader signals for L18R-AA in the presence of SDS micelles, used as a mimetic of bacterial membranes, confirming the peptide–micelle interaction with consequent variations in CSs (Figure 7B, lower spectrum). The larger CS dispersion indicates a structuration of the peptide in SDS. Hydrogen CS assignments were achieved (Table S3 and Figure S4). Unfortunately, signal broadening led to a reduction in the quality of the NMR spectra, affecting both resolution and sensitivity and precluding the assignment of ^13^C signals from the natural abundance HSQC spectrum. The differences between the H_α_ CSs in SDS and the non-structured peptide free in solution were calculated (Figure 7C). Two peptide segments show negative H_α_ shift differences (SDS-water), Leu2-Leu7 and Ala9-His14, indicating that these segments adopt the helical conformation when L18R-AA interacts with SDS micelles. The C-terminal region from Leu16 also shows negative H_α_ shift differences, suggesting that this region might populate the helical region but less stably. In addition, sequential amide cross-peaks in the NOESY spectrum confirm the stabilization of the helical conformation for L18R-AA in the presence of SDS micelles (Figure S4B).

## 3. Discussion

A significant amount of research has emerged recently about AMPs as a promising alternative or complement to conventional drugs for addressing the increasing threat of antimicrobial resistance [40,41,42,43,44].

This study focused on the rational design and characterization of novel molecules derived from the L18R peptide, which is active against yeasts and bacteria, including drug-resistant and/or biofilm-associated strains [33,34,35]. Our approach aimed at obtaining new molecules with improved activity, even against drug-resistant bacteria. In this line of research, several recent studies have reported the development of novel AMPs based on previously identified AMPs from various sources [45,46,47].

In our work, none of the newly designed peptides exhibited significant toxicity against THP-1 cells. They proved to exert, conversely, bactericidal in vitro activity against the tested staphylococcal strains, with EC_50_ values in the micromolar range (Table S1). Time-killing studies attributed fast killing kinetics to the peptide derivatives (Figure 3), except for L18R-15A. The rapid killing rates observed are consistent with a membranolytic action mechanism [48], a hypothesis further substantiated by the CLSM results (Figure 4, Figures S1 and S2). The CLSM images demonstrate a rapid loss of cell viability in a portion of the bacterial cells after only 5 min of treatment with the L18R-AA peptide, resulting from irreversible membrane permeabilization.

Following the removal of the proline residues, the L18R-AA peptide showed reduced EC_50_ values against all tested strains compared to the original L18R peptide, resulting in a 1.3 to 3.3-fold increase in antibacterial activity, particularly against the *S. aureus* reference ATCC 29213 and the MRSA strain ATCC BAA-1556. Furthermore, the substitution of the bulky residue Trp10 (L18R-10A) enhanced antibacterial activity against the resistant strains ATCC BAA-1556 (2.5-fold) and MRSA/VRSA Mu50 (1.7-fold). Instead, the exchange of Arg5 and Leu7 (L18R-LR) led to decreased antibacterial activity against all strains. Interestingly, the L18R-LR peptide adopts a β-sheet conformation in the presence of membrane mimetics, such as SDS, and LTA, a component of the cell wall of Gram-positive bacteria (Figure 5B,C).

L18R-AA, unlike the other designed peptides, has a higher aliphatic index and higher hydrophobicity than L18R (Table 1). In addition, it showed a propensity to adopt a helical conformation in the presence of TFE, LTA, and, notably, in the presence of SDS micelles (Figure 6). However, no interaction with PGN was observed. LTA primarily anchors the bacterial cell wall to the membrane via glycolipids, while PGN is the main cell wall component. This indicates that the interaction of the peptide with the bacterial cell is primarily mediated by the membrane and, to a lesser extent, by LTA. These findings indicate that the two alanine substitutions influenced the interaction of the peptide with bacterial membranes and enhanced its biological efficacy (Figure 2) [36,49,50,51,52].

We employed NMR spectroscopy to obtain residue-level information about the L18R-AA conformation in solution, both alone and in the presence of SDS micelles. The NMR data confirm the CD results: the increase in the CS dispersion of the signals in SDS is consistent with the structuration of the peptide upon the interaction with the micelles. Furthermore, the peptide in SDS adopts a helical conformation from Leu2 to His14 with a hinge at Gly8. An α-helix structure may facilitate pore formation in the bacterial cell membrane, resulting in cell death, as demonstrated by the CLMS studies (Figure 4, Figures S1 and S2). The presence of a bend or a hinge at the center of a helix in AMPs has been proposed to improve the insertion of the peptides into the lipid bilayer, facilitating their interaction with bacteria while reducing the cytotoxic effects on host cells [53,54,55].

## 4. Materials and Methods

### 4.1. Bacterial Strains

Methicillin susceptible *S. aureus* reference strains (ATCC 29213 and Newman [56]) as well as MRSA (N315 [57] and ATCC BAA-1556) and MRSA/VRSA (Mu50 [57]) strains were used in this study. Bacterial strains were maintained on Mueller–Hinton agar (MHA). Before each experiment, fresh cultures were prepared on MHA plates incubated overnight at 37 °C. The bacteria were obtained from the collection of the Laboratory of Molecular Microbiology and Biotechnology, Department of Medical Biotechnologies, University of Siena, Italy.

### 4.2. In Silico Analysis of Peptides

L18R-derived peptides were rationally designed by focusing on net charge, hydrophobicity, and amphiphilicity, using the Expasy platform from the Swiss Institute of Bioinformatics (https://www.expasy.org/ (accessed on 22 January 2025)).

### 4.3. Peptide Synthesis

The selected peptides were synthesized using the fluoren-9-ylmethoxycarbonyl (Fmoc) solid-phase synthesis chemistry, purified by HPLC, and analyzed by mass spectroscopy at the CRIBI Biotechnology Centre (University of Padua, Padua, Italy), as previously described [37]. The purity of all the investigated peptides was >98%. Mass spectra and HPLC chromatograms of the purified peptides are shown in Figure S5. The protocol used in the laboratory for testing biological activities involves the preparation of a stock solution (20 mg/mL) of peptides in dimethyl sulfoxide (DMSO), followed by proper water dilutions. All peptide solutions were stored at 4 °C. Controls (without peptides) contained DMSO at proper concentrations. To avoid the interference of DMSO, for CD experiments, peptides were solubilized in water, while for NMR, both buffer and DMSO-d_6_ were used.

### 4.4. Peptide Cytotoxicity

The cytotoxicity of the peptides was evaluated against the monocyte cell line THP-1 (ATCC TIB-202). Cells were cultured in suspension flasks in RPMI 1640 medium (Thermo Fisher Scientific, Waltham, MA, USA) supplemented with 10% heat-inactivated fetal bovine serum (FBS), 100 U/mL penicillin, and 100 μg/mL streptomycin. THP-1 cells were seeded in a 96-well plate (5 × 10^4^ cell/well) and incubated for 24 h at 37 °C in a 5% CO_2_ atmosphere. Cells were then treated with each peptide at 200, 100, 50, and 25 μM concentrations. Cell viability was assessed using the Invitrogen™ CyQUANT™ XTT Cell Viability Assay kit (Thermo Fisher Scientific Inc., Waltham, MA, USA). This assay includes the XTT reagent (2,3-Bis-(2-Methoxy-4-Nitro-5-Sulfophenyl)-2H-Tetrazolium-5-Carboxanilide), a tetrazolium-based compound sensitive to cellular redox potential, along with an Electron Coupling Reagent. Metabolically active cells convert XTT into an orange-colored formazan product, serving as an indicator of cellular respiration. Briefly, after 20 h of incubation, 6 mL of XTT reagent was added to 1 mL of Electron Coupling Reagent and mixed by vortexing. Immediately after preparation, 70 μL of this solution was added to each well. Plates were incubated for a further 4 h at 37 °C, and absorbance was measured at 450 nm and 660 nm using the Biotek Epoch 2 microplate (Agilent, Santa Clara, CA, USA). Each assay was performed in triplicate. Cells in medium without peptides and containing DMSO at proper concentrations were used as a control. The specific absorbance of each sample, which is proportional to cell viability, was calculated using the following formula: Specific Absorbance = [Abs450 nm (Test) − Abs450 nm (Blank)] − Abs660 nm (Test). The percentage of viable cells was calculated in proportion to the untreated cells.

### 4.5. Evaluation of In Vitro Antibacterial Activity of Peptides

The antibacterial activity of the peptides against *S. aureus* strains was evaluated in vitro by CFU assays, as previously described, with minor modifications [58]. Briefly, bacterial cells were cultured on MHA plates at 37 °C for 24 h and then suspended to 3 × 10^3^ cells/mL in 100 μL of sterile distilled water with each peptide at serial dilutions (50–0.2 μM). Bacterial cells in water served as controls. After 5 h at 37 °C, bacterial suspensions were plated on MHA, and colonies were counted after further incubation at 37 °C for 24 h. Per cent killing was determined relative to controls. Each assay was conducted in triplicate, and at least two independent experiments were carried out for each condition. EC_50_ values were calculated using Prism 5 software (Graph Pad, San Diego, CA, USA) through nonlinear regression analysis.

Killing kinetics of peptides were also evaluated using concentrations equal to half of their concentration values able to determine 100% killing in CFU assays. Samples for CFU determination were collected after 2, 5, 8, 10, 20, 30, and 60 min incubation at 37 °C.

### 4.6. CLSM Study

Confocal microscopy studies were performed as previously described with minor modifications [58]. Living bacterial cells, grown for 24 h at 37 °C on MHA, were resuspended in sterile water at a concentration of 3 McFarland. Subsequently, 200 µL of the suspension was centrifuged at 5000× *g* for 5 min at 20 °C, and the pellet was resuspended in 20 μL of water in the presence or absence of L18R-AA at a concentration of 100 µM. After incubation for 5 min at 37 °C, the samples were centrifuged for 5 min at 20 °C at 5000× *g*, and the bacterial cells were stained using a live/dead kit (LIVE/DEAD™ *Bac*Light™ Bacterial Viability Kit, Invitrogen, Waltham, MA, USA). Staining took place at room temperature for 20 min, protected from light. The SYTO 9 marker has a high affinity for DNA, penetrates both viable and non-viable cells, and has a maximum excitation wavelength of ~483 nm and a maximum fluorescence emission of ~503 nm (green). PI can penetrate exclusively into non-viable cells with damaged membranes, and once intercalated with DNA, it has an excitation maximum of ~535 nm and an emission maximum of ~615 nm (red). Finally, the samples were centrifuged at 5000× *g* for 5 min at 20 °C, and the pellet was resuspended in 20 µL of water with agarose (1%). The suspension was seeded on coverslips mounted in a special flow chamber for CLSM imaging. Images were acquired using a STELLARIS 5 Confocal System (Leica Microsystems, Wetzlar, Germany) integrated with a WLL laser and Diode UV Laser, combined with HyDS spectral detectors and equipped with the DMi8 inverted microscope. The scans thus obtained were processed using the LAS X program (Leica Microsystems, Wetzlar, Germany).

### 4.7. CD Studies

Far-UV CD experiments were performed using a J-1500 Circular Dichroism Spectrophotometer (Jasco, Easton, MD, USA) equipped with a Peltier thermal controller set at 20 °C. The starting peptide solutions were prepared in water (1 mM) and stored at 4 °C. CD samples were obtained by dilution to a final concentration of 50 µM and analyzed immediately or at different time points (3 weeks and 3 months after their solubilization), in water, SDS (50 mM), TFE (30%), LTA (0.02%), or PGN (0.02%). CD spectra were recorded in 0.1 cm path length quartz cuvettes ranging from 250 to 195 nm, 0.5 nm wavelength steps, a 50 nm/min scanning speed, a 1.0 nm bandwidth, and four accumulations. Following baseline correction, the observed ellipticity θ (millidegrees) was converted to molar mean residue ellipticity [θ] (deg cm^2^ dmol^−1^).

### 4.8. NMR Studies

The NMR spectra of L18R-AA were recorded at 25 °C using a 400 MHz Bruker Avance spectrometer (Bruker Corporation, Billerica, MA, USA) equipped with a 5 mm Prodigy cryoprobe and z-axis gradients, as well as a JEOL 600 MHz ECZ600R spectrometer (JEOL Inc., Tokyo, Japan) equipped with a 5 mm Royal probe and z-axis gradients. The spectra were acquired and processed using Topspin 4.3.0 (Bruker) or Delta v6.1 (JEOL) software. The recorded data were analyzed with MestReNova v14.3.3 (Mestrelab Research S.L.) or CCPNMR v3 [59].

For the NMR chemical shift assignment, a 2 mM L18R-AA sample in 50 mM phosphate buffer at pH 5.5, containing 5% D_2_O, was used. Hydrogen and carbon resonances of the reduced peptide were assigned using the following 2D NMR experiments: ^1^H-^1^H TOCSY (mixing time 80 ms), ROESY (mixing time 250 ms), and natural abundance ^1^H-^13^C HSQC [60]. The 2D spectra used watergate and dante presaturation schemes for solvent suppression. L18R-AA random coil chemical shifts were obtained from Hendus-Altenburger et al. [61]. The order parameter, S^2^, for each residue, was calculated based on backbone chemical shifts using the TALOS-N server [62].

For the structural analysis in the SDS micellar system, experiments were performed with a 2 mM L18R-AA sample (reduced form) in 50 mM phosphate buffer at pH 5.5, containing 5% D_2_O in the presence of 50 mM SDS-*d*_25._ The same set of 2D NMR experiments was used, but a NOESY spectrum (mixing time of 250 ms) was used instead of the ROESY. The spectra did not change in the presence of 100 mM SDS-*d*_25_.

## 5. Conclusions

In summary, we designed novel antimicrobial sequences that show enhanced activity against *S. aureus* compared to the parent peptide L18R. Notably, L18R-AA demonstrates strong efficacy against all tested *S. aureus* strains, including the drug-resistant isolates. L18R-AA interacts with bacterial membrane mimetics, stabilizing a helical conformation. The investigation of other substitutions may help increase our understanding of the role of individual residues, potentially leading to the development of even more potent and less toxic derivatives. The next step in this perspective could be a further derivative peptide combining the substitutions made for L18R-AA and L18R-10A, which exhibited the highest activity against most of the tested strains. The future roadmap will also include other experimental studies to deepen the knowledge on the exact mechanism of action of the described peptides. Moreover, a fundamental step will be the evaluation of the therapeutic potential of the selected molecules in experimental models of infection. Overall, L18R-AA may offer a foundation for further studies to develop novel therapeutic approaches to combat infections caused by drug-resistant bacteria.

## Figures and Tables

**Table 1:** Amino acid sequences and characteristics of investigated peptides.

Peptide	Sequence	MM (Da)	pI	Charge	AI	GRAVY
**L18R**	**LLVLRSLGPWHPGHCLLR**	**2067.53**	**10.35**	**2+**	**146.11**	**0.467**
L18R-AA	LLVLRSLG**A**WH**A**GHCLLR	2015.45	10.35	2+	157.22	0.844
L18R-10A	LLVLRSLGP**A**HPGHCLLR	1952.39	10.35	2+	151.67	0.617
L18R-15A	LLVLRSLGPWHPGH**A**LLR	2035.47	12.00	2+	151.67	0.428
L18R-LR	LLVL**L**S**R**GPWHPGHCLLR	2067.53	10.35	2+	146.11	0.467

MM (Da), molecular mass (Dalton); pI, isoelectric point; AI, aliphatic index; GRAVY, grand average of hydropathy. In bold is the parental peptide (first row) and the substituted residues (in red). MM, pI, charge, AI, and GRAVY were calculated using the ExPASy tool ProtParam.

## Data Availability

The original contributions presented in this study are included in the article/Supplementary Material. Further inquiries can be directed to the corresponding author.

## Supplementary Materials

The following supporting information can be downloaded at https://www.mdpi.com/article/10.3390/antibiotics14050437/s1, Table S1: In vitro antibacterial activity of the investigated peptides against planktonic cells of *Staphylococcus aureus;* Figure S1: CLSM images of viable *S. aureus* ATCC 29213 cells treated with 100 µM L18R-AA; Figure S2: CLSM images of viable *S. aureus* ATCC BAA-1556 cells treated with 100 µM L18R-AA; Figure S3: Expansions of the ^1^H-^13^C HSQC multiplicity edited spectrum of reduced L18R-AA; Figure S4: Amide region of (A) ROESY spectrum of reduced L18R-AA in buffer, (B) NOESY spectrum in SDS-*d*_25_, (C) amide-alpha region of ^1^H-^1^H TOCSY, and (D) NOESY spectra showing sequential CS assignment ofL18R-AA in presence of SDS micelles; Table S2: CS assignment for L18R-AA in its reduced form for a 2 mM solution in 50 mM phosphate buffer at pH 5.5 at 25 °C; Table S3. CS assignment for L18R-AA in its reduced form for a 2 mM solution in 50 mM phosphate buffer at pH 5.5 in the presence of 50 mM SDS-d25 at 25 °C; Figure S5: Mass spectra and HPLC chromatograms of the purified peptides.

## Abbreviations

The following abbreviations are used in this manuscript:

AI	Aliphatic index
AMPs	Antimicrobial peptides
AMR	Antimicrobial resistance
CD	Circular dichroism
CFU	Colony-forming unit
CLSM	Confocal laser scanning microscopy
CS	Chemical shift
Da	Dalton
DMSO	Dimethyl sulfoxide
DMSO-*d*_6_	Deuterated DMSO
EC_50_	Half-maximal effective concentration
Fmoc	Fluoren-9-ylmethoxycarbonyl
GRAVY	Grand average of hydropathy
HIV	Human immunodeficiency virus
IGHJ2	Immunoglobulin heavy chain J gene
LTA	Lipoteichoic acid
MHA	Mueller–Hinton agar
MM	Molecular mass
MRSA	Methicillin-resistant *Staphylococcus aureus*
NMR	Nuclear magnetic resonance spectroscopy
PGN	Peptidoglycan
pI	Isoelectric point
PI	Propidium iodide
SDS	Sodium dodecyl sulphate
SDS-*d*_25_	Deuterated SDS
TFE	2,2,2-Trifluoroethanol
VRSA	Vancomycin-resistant *Staphylococcus aureus*
WHO	World Health Organization
