# Supplementary material for: Anti-Staphylococcus aureus Activity and Structural Characterization of Rationally Designed Peptides
Source: Antibiotics (Basel). 2025 Apr 26;14(5):437. doi: 10.3390/antibiotics14050437 (PMC12108160; doi:10.3390/antibiotics14050437)
Supplement: Supplementary file 1 [file antibiotics-14-00437-s001.zip › antibiotics-3568290-supplementary.pdf]

Table S1. In vitro antibacterial activity of the investigated peptides against planktonic cells of *Staphylococcus aureus*.

***S. aureus* strain**	**EC_{50} (95% confidence intervals) mol/L × 10^{-6}**				
	**L18R**	**L18R-AA**	**L18R-10A**	**L18R-15A**	**L18R-LR**
**ATCC 29213**	0.560 (0.357 – 0.878)	0.168 (0.089 – 0.319)	0.706 (0.563 – 0.885)	3.177 (2.713 – 3.721)	2.238 (1.908 – 2.626)
**Newman**	1.416 (1.148 – 1.747)	0.731 (0.616 – 0.868)	1.231 (0.998 – 1.518)	12.21 (8.514 – 17.51)	3.280 (3.088 – 3.485)
**ATCC BAA-1556***	1.667 (1.536 – 1.809)	0.662 (0.586 – 0.748)	0.664 (0.590 – 0.748)	2.448 (2.207 – 2.716)	2.316 (2.071 – 2.590)
**N315***	1.611 (1.427 – 1.818)	1.240 (0.738 – 2.083)	1.440 (0.969 – 2.141)	1.150 (0.842 – 1.571)	2.035 (1.750 – 2.365)
**Mu50***	2.229 (1.663 – 2.989)	1.432 (1.306 – 1.570)	1.319 (1.198 – 1.453)	18.05 (12.12 – 26.88)	3.357 (2.577 – 4.374)

EC_{50}, half-maximal effective concentration. *drug-resistant strain.

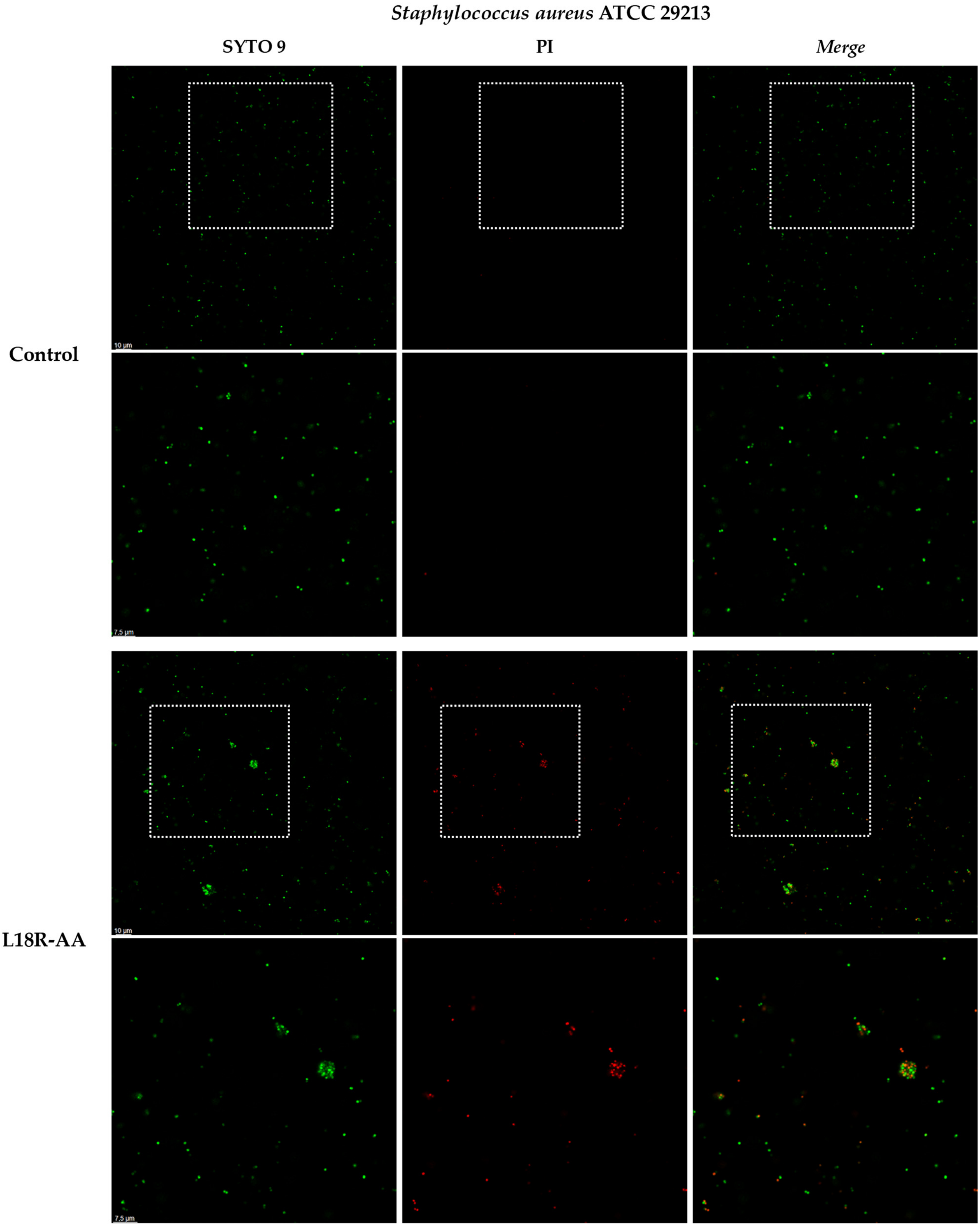

Figure S1. CLSM images of viable *S. aureus* ATCC 29213 cells treated with 100 µM L18R-AA. Bacterial cells were labeled with SYTO 9 and propidium iodide (PI) after 5 min of treatment with or without (control) the peptide. Green fluorescence: viable cells; red fluorescence: dead cells. A detail of the field highlighted by dotting is shown at higher magnification in the row below. Bars, 10 and 7.5 µm, respectively.

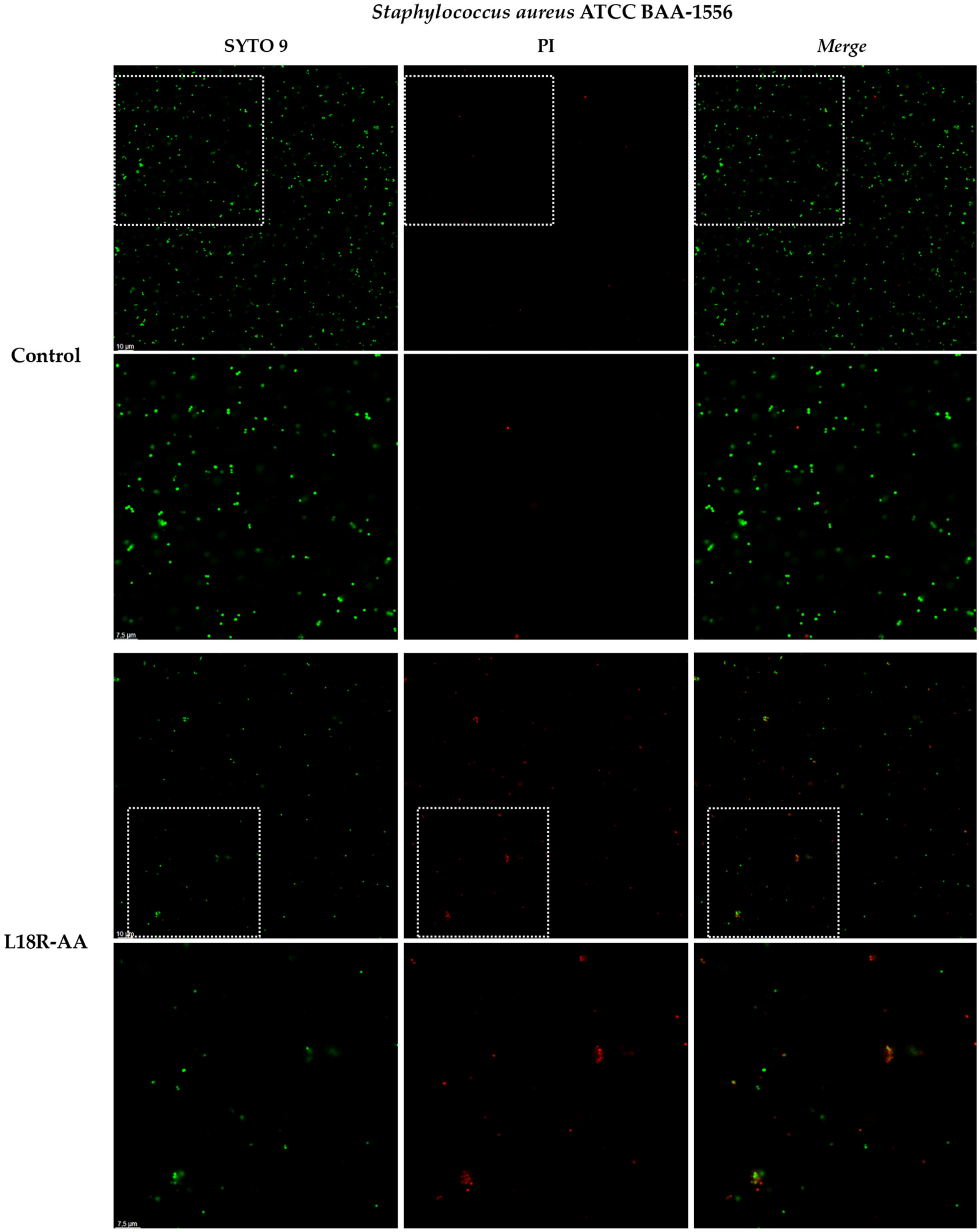

Figure S2. CLSM images of viable *S. aureus* ATCC BAA-1556 cells treated with 100 μM L18R-AA. Bacterial cells were labeled with SYTO 9 and propidium iodide (PI) after 5 min of treatment with or without (control) the peptide. Green fluorescence: viable cells; red fluorescence: dead cells. A detail of the field highlighted by dotting is shown at higher magnification in the row below. Bars, 10 and 7.5 μm, respectively.

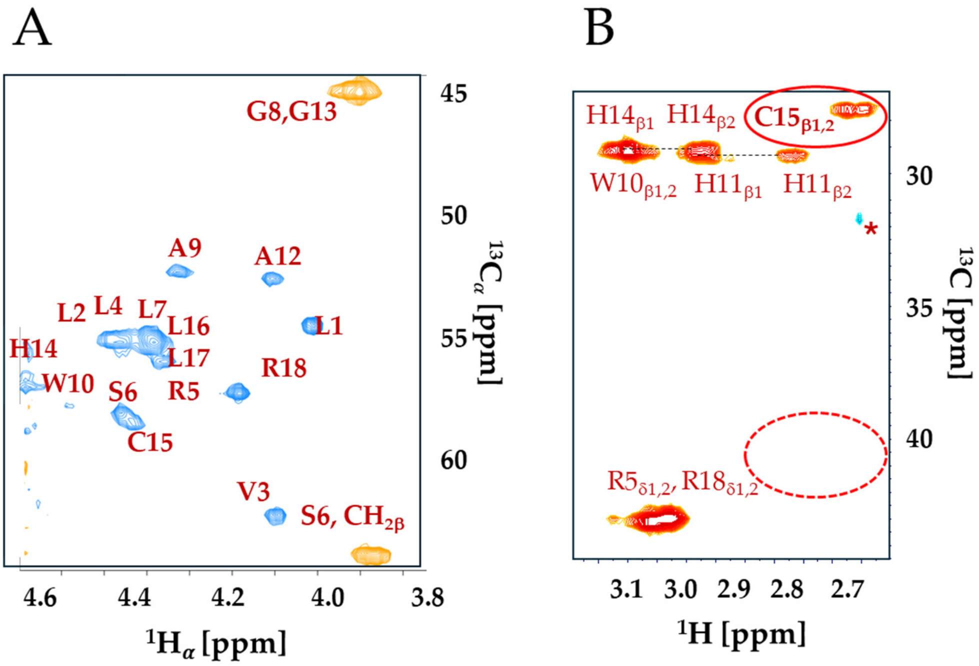

Figure S3. Expansions of the ^{1}H-^{13}C HSQC multiplicity edited spectrum of reduced L18R-AA. (A) alpha signals and (B) selected region showing the Cys15 ^{13}CHβ beta signals. The chemical shift assignments are indicated. In (B), the cysteine ^{13}Cβ signals, characteristic of the oxidation state of cysteine residues, are indicated in bold (reduced form) and circled in red (oxidized form, not present). * indicates buffer impurities.

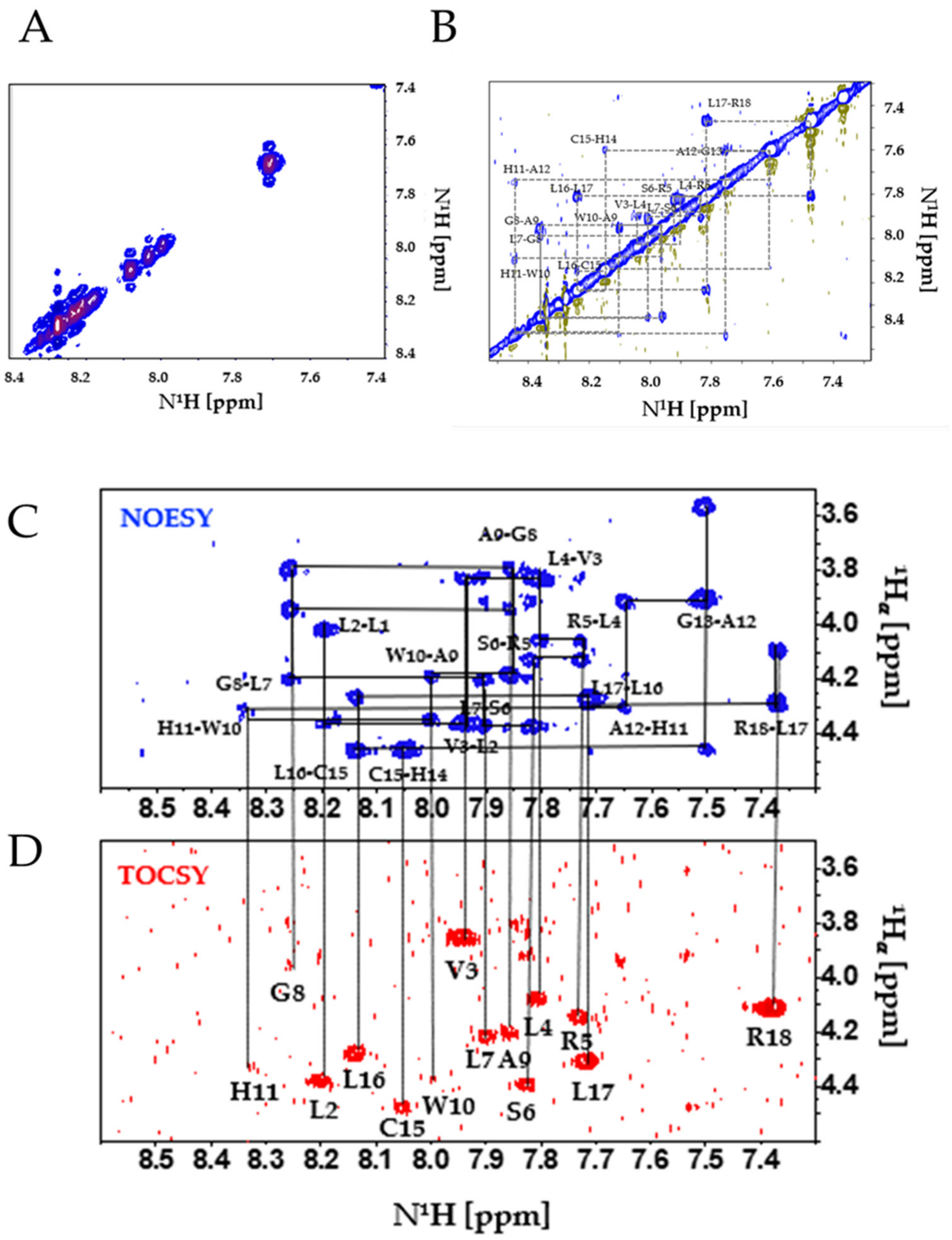

Figure S4. (A) Amide region of the ROESY spectrum of reduced L18R-AA. The lack of sequential NH-NH correlations supports that L18R-AA in solution adopts a flexible and predominantly extended conformation. (B) Amide proton region of the NOESY spectrum of L18R-AA in the presence 50 mM SDS-d_{25}. The sequential $NH_i \rightarrow NH_{i+1}$ cross-peaks, characteristic of helical conformations, are indicated. Amide-alpha region of the ^{1}H-^{1}H NOESY (C) and TOCSY (D) spectra showing the sequential assignment of L18R-AA in the presence SDS-d_{25}. This selected region shows the alpha hydrogen signals in the F1 dimension, while amide hydrogen signals are in F2. Residues are labeled at the positions of the intra-residual HN-Hα peaks in the TOCSY spectrum, while the inter-residual HN-Hα NOE connectivities are indicated in the NOESY spectrum.

Table S2. CS assignment for L18R-AA in its reduced form for a 2 mM solution in 50 mM phosphate buffer pH 5.5 at 25° C. CS values in ppm.

Residue	NH	H_α	H_β	Others
**L1**	-	4.02 (54.5)	1.69, 1.74 (42.4)	H_γ: 1.62 (26.2); $H_{\delta1}$, $H_{\delta2}$: 0.90 (24.5), 0.90 (23.9)
**L2**	8.63	4.36 (55.1)	1.56, 1.60 (42.4)	H_γ: 1.60 (26.7); $H_{\delta1}$, $H_{\delta2}$: 0.92 (24.7), 0.94 (24.1)
**V3**	8.36	4.09 (62.1)	2.02 (32.8)	$H_{\gamma1}$, $H_{\gamma2}$: 0.93 (20.4), 0.90 (20.5)
**L4**	8.40	4.40 (55.0)	1.63, 1.54 (42.2)	H_γ: 1.62 (26.5); $H_{\delta1}$, $H_{\delta2}$: 0.93 (24.0), 0.83 (23.3)
**R5**	8.37	4.36 (56.0)	1.75, 1.84 (30.7)	H_γ: 1.58, 1.58 (27.1); H_δ: 3.16, 3.16 (43.2); H_ε: 7.18
**S6**	8.31	4.47 (58.1)	3.87, 3.87 (63.3)	
**L7**	8.35	4.39 (55.1)	1.68, 1.63 (42.2)	H_γ: 1.64 (26.3); $H_{\delta1}$, $H_{\delta2}$: 0.90 (24.0), 0.89 (23.3)
**G8**	8.34	3.90, 3.90 (45.2)		
**A9**	8.14	4.32 (52.3)	1.32 (19.1)	
**W10**	8.16	4.58 (57.7)	3.22, 3.22 (29.6)	$H_{\delta1}$: 7.22 (127.3); $H_{\varepsilon1}$: 10.1; $H_{\varepsilon3}$: 7.48 (120.7); $H_{\eta2}$: 7.23 (124.6); $H_{\zeta2}$: 7.48 (114.6); $H_{\zeta3}$: 7.13 (122.0)
**H11**	8.28	4.69 (55.2)	3.09, 3.23 (29.4)	$H_{\delta1}$: 7.18 (119.9); $H_{\varepsilon1}$: 8.59 (136.7)
**A12**	8.12	4.11 (52.7)	1.34 (19.0)	
**G13**	8.33	3.90, 3.90 (45.2)		
**H14**	8.28	4.69 (55.8)	3.23, 3.09 (29.4)	$H_{\delta1}$: 7.19 (119.9); $H_{\varepsilon1}$: 8.59 (136.7)
**C15**	8.40	4.43 (58.5)	2.85, 2.85 (27.8)	
**L16**	8.41	4.37 (55.2)	1.61, 1.61 (42.4)	H_γ: 1.62 (27.0); $H_{\delta1}$, $H_{\delta2}$: 0.92 (25.0), 0.86 (23.3)
**L17**	8.21	4.38 (55.4)	1.62, 1.62 (42.2)	H_γ: 1.62 (26.6); $H_{\delta1}$, $H_{\delta2}$: 0.92 (24.9), 0.87 (23.6)
**R18**	7.84	4.18 (57.3)	1.85, 1.71 (31.7)	H_γ: 1.57, 1.57 (27.2); H_δ: 3.17, 3.17 (43.3); H_ε: 7.18

Table S3. CS assignment for L18R-AA in its reduced form for a 2 mM solution in 50 mM phosphate buffer pH 5.5 in the presence of 50 mM SDS-d25 at 25° C. CS values in ppm. The detected ^{13}C signals are reported in brackets.

Residue	NH	Hα	Hß	Others
**L1**	-	4.02 (54.6)	1.69, 1.71 (42.4)	H_{γ}: 1.68 (27.0); $H_{\delta 1}$, $H_{\delta 2}$: 0.90 (24.6), 0.90 (25.1)
**L2**	8.29	4.36	1.57, 1.66 (42.3)	H_{γ}: 1.56 (26.8); $H_{\delta 1}$, $H_{\delta 2}$: 0.83 (24.1), 0.83 (25.2)
**V3**	8.04	3.83	2.10	$H_{\gamma 1}$, $H_{\gamma 2}$: 0.85 (21.2), 0.90 (21.4)
**L4**	7.90	4.05	1.65, 1.74 (42.5)	H_{γ}: 1.58 (26.7); $H_{\delta 1}$, $H_{\delta 2}$: 0.88 (24.3), 0.80 (23.3)
**R5**	7.83	4.12	1.77, 1.85	H_{γ}: 1.60, 1.64; H_{δ}: 3.11, 3.11; He: 7.04
**S6**	7.92	4.37	3.82, 3.91	
**L7**	8.01	4.20	1.72, 1.78	H_{γ}: 1.57 (26.9); $H_{\delta 1}$, $H_{\delta 2}$: 0.76 (23.6), 0.84 (25.8)
**G8**	8.36	3.78, 3.93		
**A9**	7.96	4.18	1.40 (18.2)	
**W10**	8.10	4.34	3.28, 3.35	$H_{\delta 1}$: 7.15; $H_{\varepsilon 1}$: 9.72; $H_{\varepsilon 3}$: 7.37; $H_{\eta 2}$: 7.13; $H_{\zeta 2}$: 6.88; $H_{\zeta 3}$: 6.88
**H11**	8.44	4.30	3.09, 3.26	$H_{\delta 1}$: 7.22; $H_{\varepsilon 1}$: 8.63
**A12**	7.75	3.91	1.30 (18.4)	
**G13**	7.61	3.55, 3.55		
**H14**	7.60	4.45	2.28, 2.77	$H_{\delta 1}$: 7.03; $H_{\varepsilon 1}$: 8.28
**C15**	8.14	4.45	2.78, 2.78 (27.9)	
**L16**	8.23	4.26	1.56, 1.67 (42.3)	H_{γ}: 1.67 (26.9); $H_{\delta 1}$, $H_{\delta 2}$: 0.84 (23.8), 0.91 (25.3)
**L17**	7.81	4.28	1.54, 1.64 (42.1)	$H_{\delta 1}$, $H_{\delta 2}$: 0.83 (25.3), 0.80 (23.5)
**R18**	7.47	4.10 (56.7)	1.64, 1.80 (31.3)	H_{γ}: 1.51, 1.51 (27.0); H_{δ}: 3.10, 3.10 (43.5); H_{ε}: 6.99

LLVLRSLGPWHPGHCLLR (L18R)
Mw: 2066,162 Da

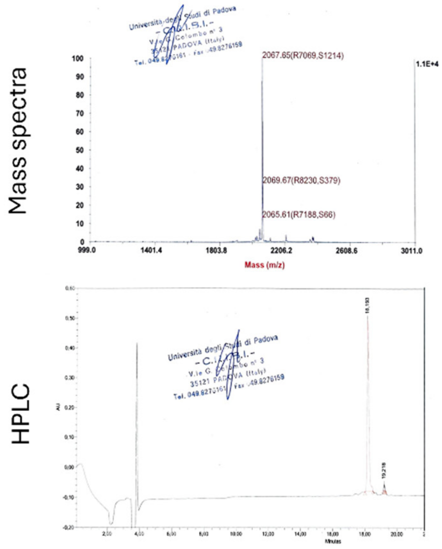

#	Retention Time (min.)	Area	% Area	Height
1	18,193	4299058	**98,06**	577924
3	19,218	84959	1,94	19700

LLVLRSLGAWHAGHCLLR (L18R-AA)
Mw: 2014,130 Da

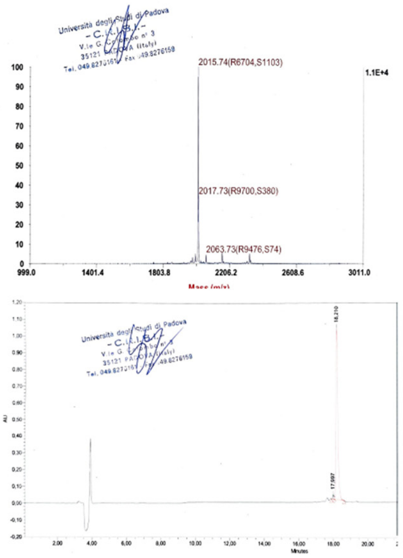

#	Retention Time (min.)	Area	% Area	Height
1	17,997	153261	2,00	26138
2	18,210	7525619	**98,00**	1026631

LLVLRSLGPAHPGHCLLR (L18R-10A)
Mw: 1951,119 Da

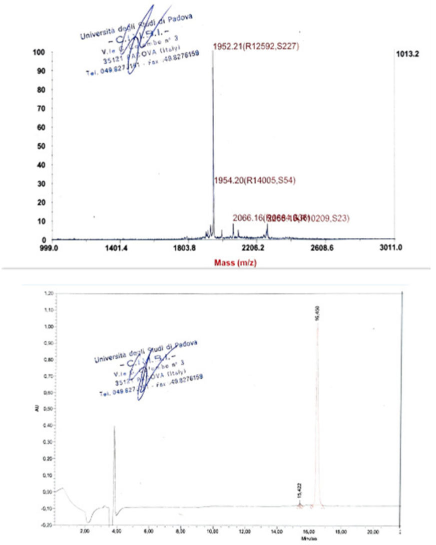

#	Retention Time (min.)	Area	% Area	Height
1	16,450	9924371	**99,26**	1081875
2	15,422	74449	0,74	11192

LLVLRSLGPWHPGHALLR (L18R-15A)
Mw: 2034,190 Da

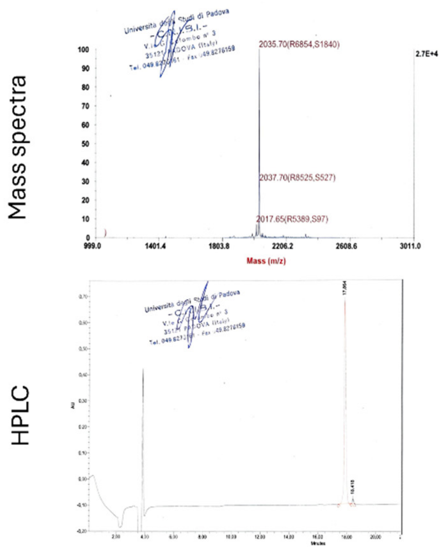

#	Retention Time (min.)	Area	% Area	Height
1	17,864	6245142	**98,18**	778967
2	18,418	115753	1,82	16605

LLVLLSRGPWHPGHCLLR (L18R-LR)
Mw: 2066,162 Da

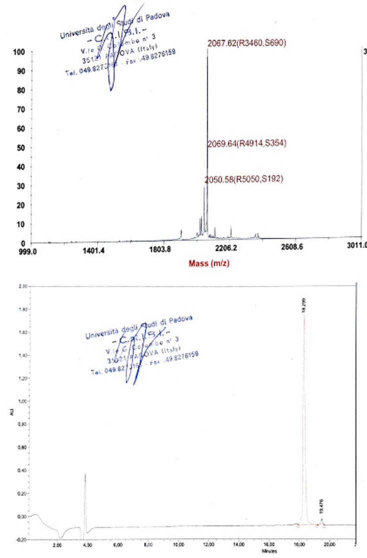

#	Retention Time (min.)	Area	% Area	Height
1	18,299	17697153	**98,01**	1801379
2	19,476	359493	1,99	47955

Figure S5. Mass spectra and HPLC chromatograms of purified peptides.
